# Femtosecond Laser Arcuate Keratotomy vs Toric Intraocular Lens Implantation in Cataract Surgery: A Randomized Clinical Trial

**DOI:** 10.1001/jamaophthalmol.2024.5887

**Published:** 2025-01-23

**Authors:** Yueyang Zhong, Shuying Chen, Hanle Wang, Su Li, Zhouyu Lu, Jingjie Xu, Yibo Yu, Ke Yao

**Affiliations:** 1 Eye Center, The Second Affiliated Hospital, School of Medicine, Zhejiang University, Zhejiang Provincial Key Laboratory of Ophthalmology, Zhejiang Provincial Clinical Research Center for Eye Diseases, Zhejiang Provincial Engineering Institute on Eye Diseases, Hangzhou, China

## Abstract

**Importance:**

After cataract surgery, postoperative residual astigmatism can influence a patient’s visual quality and satisfaction. Finding ways to minimize this astigmatism is important.

**Objective:**

To compare the clinical outcomes of femtosecond laser arcuate keratotomy (FSAK) and toric intraocular lens (TIOL) implantation for astigmatism correction in patients undergoing femtosecond laser-assisted cataract surgery.

**Design, Setting, and Participants:**

This randomized clinical trial was conducted between October 2021 and September 2023 at the Eye Center of the Second Affiliated Hospital, School of Medicine, Zhejiang University, China. Patients with mild to moderate regular corneal astigmatism ranging from 0.75 diopters (D) to 3.00 D were recruited.

**Interventions:**

Patients were randomly assigned in a 1:1 ratio to receive FSAK or TIOL implantation.

**Main Outcomes and Measures:**

The primary outcome was the subjective manifest refraction assessed at 3 months postoperatively.

**Results:**

A total of 196 patients (mean [SD] age, 68.4 [13.7] years; 124 female [63%]) were randomized to the FSAK group (98 [50%]) or TIOL group (98 [50%]), and 92 patients (94%) and 95 patients (97%), respectively, finished the 3-month follow-up. At 3 months postoperatively, there was no difference in the refractive astigmatism between the 2 groups (mean [SD], 0.64 [0.64] D vs 0.54 [0.55] D; difference, 0.11 D; 95% CI, −0.06 to 0.27 D; *P* = .21). The mean (SD) uncorrected distance visual acuity was 0.15 (0.20) logMAR (Snellen equivalent, 20/28) for the FSAK group and 0.14 (0.19) logMAR (Snellen equivalent, 20/28) for the TIOL group (difference, 0.01 D; 95% CI, −0.04 to 0.06 D; *P* = .71). Subgroup analyses showed that TIOL implantation achieved lower residual astigmatism compared with FSAK when treating astigmatism exceeding 1.5 D or against-the-rule astigmatism.

**Conclusions and Relevance:**

Results of this randomized clinical trial demonstrate that in study participants with mild to moderate astigmatism undergoing femtosecond laser-assisted cataract surgery, FSAK was not superior to TIOL implantation for astigmatism correction. However, for astigmatism exceeding 1.5 D or against-the-rule astigmatism, TIOL implantation demonstrated greater efficacy over FSAK. Future trials are needed to determine if there is greater efficacy of TIOL implantation over FSAK as suggested by subgroup analyses.

**Trial Registration:**

ChiCTR.org.cn Identifier: ChiCTR2100051066

## Key Points

**Question:**

Is femtosecond laser arcuate keratotomy (FSAK) superior to toric intraocular lens (TIOL) implantation to correct astigmatism in patients undergoing femtosecond laser-assisted cataract surgery (FLACS)?

**Findings:**

In this randomized clinical trial that included 196 patients, FSAK was not superior to TIOL implantation for astigmatism correction. Subgroup analyses suggested that TIOL implantation achieved lower residual astigmatism when astigmatism exceeded 1.5 diopters or against-the-rule astigmatism.

**Meaning:**

Although FSAK was not superior to TIOL implantation for astigmatism correction, future trials are needed to determine if there is greater efficacy of TIOL implantation over FSAK as suggested by subgroup analyses.

## Teaser

This randomized clinical trial investigates if femtosecond laser arcuate keratotomy is superior to toric intraocular lens implantation to correct astigmatism in patients undergoing femtosecond laser-assisted cataract surgery.

## Introduction

With decades of advancements and innovations in ophthalmology, cataract surgery has undergone a paradigm shift to refractive surgery in this modern era. Nevertheless, residual astigmatism continues to be one of the major factors influencing patients’ visual quality and satisfaction.^1^ Notably, when the corneal astigmatism is greater than 0.75 diopters (D), visual blurring, fatigue, glare, and diplopia may occur, and patients are unlikely to achieve spectacle independence.^2,3,4^ Clinical evidence has suggested that reducing astigmatism to less than 0.5 D helps to achieve optimal visual function and patient satisfaction.^2,5^

To date, several approaches have been developed to address preoperative astigmatism in cataract surgery, including clear corneal incisions, limbal relaxing incisions, arcuate keratotomy, and toric intraocular lens (TIOL) implantation, each with demonstrated efficacy.^1,3,6,7^ Among various approaches, TIOL implantation has been extensively studied and clinically practiced for astigmatism correction.^1,3,8,9^ Recent studies^3,4,10^ have highlighted the superior efficiency of TIOL implantation compared with other incisional methods. Although manual arcuate keratotomy has been used clinically for decades, it is often associated with decreased precision and predictability.^10,11^ Recently, femtosecond laser arcuate keratotomy (FSAK) has emerged as a superior alternative to manual arcuate keratotomy, offering improved precision and consistency and has been proven to be an effective approach for astigmatism correction.^11,12,13,14,15^

As both TIOL implantation and FSAK are effective methods for addressing astigmatism, there has been much interest in comparing their efficacy in cataract surgery. Previous studies have compared the refractive outcomes of patients undergoing femtosecond laser-assisted cataract surgery (FLACS) with FSAK and those undergoing conventional phacoemulsification with TIOL implantation.^16,17,18,19,20^ Despite variations in the FSAK nomograms across these studies, most have reported comparable results between the 2 approaches.^16,18,19,20^ However, these studies were limited by nonrandomized designs and relatively small sample sizes. Additionally, for TIOL implantation, conventional manual capsulotomy was less precise compared with femtosecond laser-assisted capsulotomy, which can create a well-centered and accurately sized opening for IOL implantation.^21,22^ Therefore, we conducted a randomized clinical trial exclusively involving patients receiving FLACS to compare these 2 procedures for astigmatism correction.

## Methods

### Study Design

This 2-group, parallel, randomized clinical trial was performed at the Eye Center of the Second Affiliated Hospital, School of Medicine, Zhejiang University, China. The study protocol is available in Supplement 1, and the statistical analysis plan is available in Supplement 2. The protocol received approval from the institutional review board of the hospital and adhered to the principles of the Declaration of Helsinki. The patients provided written informed consent before randomization and received no stipend or other incentives. The trial was registered and the results were reported following the Consolidated Standards of Reporting Trials (CONSORT) reporting guidelines.

### Eligibility Criteria

Patients with cataract who were scheduled for FLACS with IOL implantation were assessed for eligibility between October 2021 and September 2023. Inclusion criteria included patients aged from 18 to 80 years with mild to moderate regular corneal astigmatism ranging from 0.75 D to 3.0 D. The patients were asked for medical records such as race and ethnicity. All patients enrolled were Chinese and of Han ethnicity. Exclusion criteria included the following: (1) ocular surface abnormalities such as irregular corneal astigmatism, corneal scarring, keratoconus, and pterygium; (2) history of ocular trauma or surgery; (3) presence of coexisting ocular disorders such as glaucoma, retinal vascular occlusive disease, retinal detachment, diabetic retinopathy, and any optic nerve-related pathologies; (4) poorly dilated pupil with a diameter less than 5.0 mm; (5) concurrent severe systemic diseases; and (6) lack of cooperation. When both eyes of a patient met the eligibility criteria, only 1 eye was enrolled in the study.

### Randomization

Eligible patients were randomly allocated into 2 groups: the FSAK group or the TIOL group. A statistician who had no contact with the patients generated a computer-based random-number table with an allocation ratio of 1:1. Group allocation was concealed in sequentially numbered opaque envelopes. Neither the patients nor the surgeon was masked to the interventions. The examiners responsible for outcome assessments and data screening were blinded to the allocation throughout the study period.

### Surgical Technique

All surgical procedures were performed by the same surgeon (K.Y.) using a femtosecond laser platform (LenSx [Alcon Laboratories]) and a phacoemulsification system (Centurion [Alcon Surgical]). Details of FLACS have been previously reported and are summarized in the eMethods in Supplement 3. Full correction of astigmatism was aimed in all cases.

In the FSAK group, the femtosecond laser was used to perform anterior capsulotomy and lens fragmentation, followed by the FSAK procedure. Symmetrical paired corneal arcuate keratotomies were performed at an 8.5-mm diameter optical zone, with a depth of 90% corneal pachymetry. The arc length and position of the arcuate incisions were determined by the modified Donnenfeld nomogram, using an online calculator.^23^ Considering that the diameter of the arcuate incisions was 11.0 mm, geometric modification was applied to adjust the arc length, resulting in a corrected arc length of 8.5/11 of the original value. Following FLACS surgery, a monofocal IOL (Tecnis ZCB00 [Johnson & Johnson Vision]) was implanted, and the arcuate incisions were dissected with a blunt spatula to ensure complete separation.

In the TIOL group, the TIOL power and alignment axis were calculated using an online calculator.^24^ Intraoperatively, the intended implantation axis was marked on the limbus by aligning a Mendez ring with the horizontal marks. Following FLACS surgery, a TIOL (Tecnis Toric ZCT [Johnson & Johnson Vision]) was implanted and adjusted to its final targeted position by aligning the toric reference marks with the limbal axis marks.

### Outcome Measures

The primary outcome was the refractive astigmatism measured by subjective refraction examination at 3 months postoperatively. The secondary outcomes included uncorrected distance visual acuity (UDVA), corrected distance visual acuity (CDVA), and corneal topography assessments. Visual acuity was measured using the standardized logarithmic visual acuity chart (GB11533-2011) with E optotypes and converted into logMAR units.^25^ Any intraoperative and postoperative adverse events were documented.

### Statistical Analysis

The sample size was calculated based on the primary outcomes and the results from a previous study.^20^ To provide 90% power to detect a difference of refractive astigmatism of 0.05 D with a 2-sided significance level of .05, a sample size of 196 (98 in each group) was estimated with consideration of a dropout rate of 20%. The sample size calculation was performed using PASS, version 16.0 (NCSS).

All analyses were performed on an intention-to-treat (ITT) basis. Missing data were implemented through multiple imputation technique with details provided in the eMethods in Supplement 3. Post hoc subgroup analyses were performed based on the magnitude of preoperative astigmatism and the astigmatism type (eMethods in Supplement 3). Vector analysis, which is a standard method for astigmatic analysis by the American National Standards Institute, was performed using the Alpins method as defined in the eMethods in Supplement 3.^26,27,28^

Variable normality was assessed using the Shapiro-Wilk test. The 2-sample *t* test was used for comparing continuous variables between groups, and Pearson χ^2^ test was used for categorical variables. Statistical significance was defined based on 2-sided tests with *P* <.05 for the primary outcome. There was no adjustment for multiple analyses for secondary outcomes or other outcomes. Data analysis was conducted using SPSS, version 26.0 (IBM), and R, version 3.6.2 (R Foundation for Statistical Computing).

## Results

### Participant Characteristics

**Figure 1.**
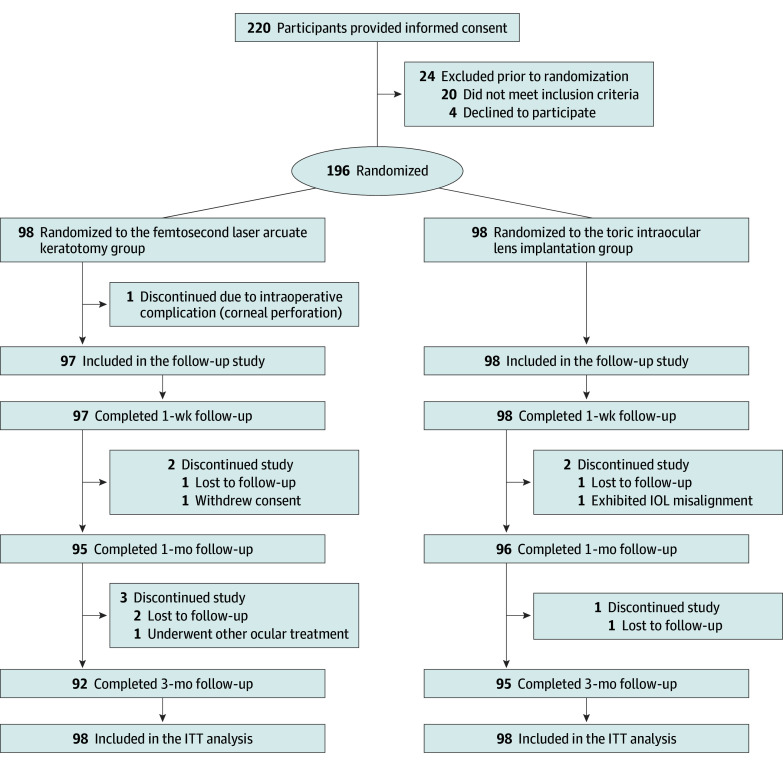
Flow Diagram of the Enrollment and Follow-Up Process IOL indicates intraocular lens; ITT, intention to treat.

Between October 2021 and September 2023, a total of 220 patients with age-related cataract were screened for eligibility. Among them, 24 (11%) were deemed ineligible, and the remaining 196 patients (mean [SD] age, 68.4 [13.7] years; 124 female [63%]; 72 male [37%]) were randomized into the FSAK group (98 [50%]) or the TIOL group (98 [50%]). All patients enrolled were Chinese and of Han ethnicity (196 [100%]). During the follow-up period, 9 patients missed follow-up examination, 5 refused to continue the study, 2 exhibited adverse events related to the intervention, and 2 underwent other ocular treatment (Figure 1). A total of 92 patients (94%) and 95 patients (97%) in the FSAK and TIOL groups, respectively, completed the follow-up at 3 months. The baseline characteristics of the study population are provided in Table 1. There were 61 patients (62%) and 65 patients (66%) with preoperative astigmatism exceeding 1.5 D in the FSAK and TIOL groups, respectively. Both groups exhibited comparable distributions of with-the-rule (WTR), against-the-rule (ATR), and oblique astigmatism. A total of 56 patients (57%) in the FSAK group and 54 patients (55%) in the TIOL group had ATR astigmatism.

**Table 1.** Baseline Characteristics

Parameters	FSAK (n = 98)	TIOL (n = 98)
Age, mean (SD), y	68.5 (13.1)	68.2 (14.2)
Sex, No. (%)		
Female	63 (64)	61 (62)
Male	35 (36)	37 (38)
Eye laterality, No. (%)		
Right	42 (43)	49 (50)
Left	56 (57)	49 (50)
Refractive astigmatism, mean (SD), D	1.56 (0.77)	1.67 (0.77)
Keratometric astigmatism, mean (SD), D	1.40 (0.51)	1.56 (0.63)
UDVA		
LogMAR, mean (SD)	0.81 (0.50)	0.74 (0.53)
Snellen equivalent, mean	20/130	20/110
CDVA		
LogMAR, mean (SD)	0.37 (0.26)	0.33 (0.24)
Snellen equivalent, mean	20/46	20/43
Preoperative astigmatism, No. (%)		
<1.5 D	37 (38%)	33 (34%)
≥1.5 D	61 (62%)	65 (66%)
Astigmatism type, No. (%)		
WTR	29 (30)	30 (31)
ATR	56 (57)	54 (55)
Oblique	13 (13)	14 (14)

Abbreviations: ATR, against the rule; CDVA, corrected distance visual acuity; D, diopter; FSAK, femtosecond laser arcuate keratotomy; TIOL, toric intraocular lens; UDVA, uncorrected distance visual acuity; WTR, with the rule.

### Refractive Astigmatism

**Figure 2.**
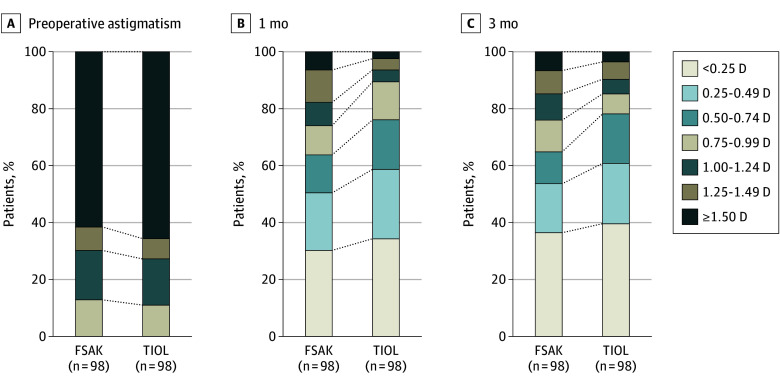
Distribution of Refractive Astigmatism Preoperatively and Postoperatively in the Femtosecond Laser Arcuate Keratotomy (FSAK) and Toric Intraocular Lens (TIOL) Groups Each cell corresponds to an astigmatism range on the scale, with the width of each cell representing the percentage of study participants within that range. D indicates diopter.

Preoperatively, there was no difference in the preoperative astigmatism between the FSAK and TIOL groups (mean [SD], 1.56 [0.77] D vs 1.67 [0.77] D; difference, −0.11 D; 95% CI, −0.32 to 0.10 D; *P* = .32). As shown in Table 2, the mean (SD) refractive astigmatism was 0.64 (0.64) D for the FSAK group and 0.54 (0.55) D for the TIOL group (difference, 0.11 D; 95% CI, −0.06 to 0.27 D; *P* = .21) at 3 months postoperatively. There were also no differences between groups at other follow-up visits (eTable 1 in Supplement 3). The postoperative distribution of refractive astigmatism showed that more patients in the TIOL group maintained residual astigmatism less than 0.75 D compared with the FSAK group, at both 1 month and 3 months postoperatively (Figure 2). In the double-angle plots, preoperative refractive astigmatism points were scattered but became more concentrated at 3 months postoperatively in both groups (eFigure 1 in Supplement 3).

**Table 2.** Primary and Key Secondary Outcomes at 3 Months

Outcomes	FSAK (n = 98)	TIOL (n = 98)	Mean difference (95% CI)	*P* value^a^
Primary outcome				
Refractive astigmatism, mean (SD), D	0.64 (0.64)	0.54 (0.55)	0.11 (−0.06 to 0.27)	.21
**Key secondary outcomes**				
UDVA				
LogMAR, mean (SD)	0.15 (0.20)	0.14 (0.19)	0.01 (−0.04 to 0.06)	.71
Snellen equivalent, mean	20/28	20/28		
CDVA				
LogMAR, mean (SD)	0.07 (0.16)	0.07 (0.14)	0.004 (−0.04 to 0.05)	.82
Snellen equivalent, mean	20/23	20/23		
Keratometric astigmatism, mean (SD), D	0.90 (0.64)	1.43 (1.08)	−0.53 (−0.82 to −0.24)	.002

Abbreviations: CDVA, corrected distance visual acuity; D, diopter; FSAK, femtosecond laser arcuate keratotomy; TIOL, toric intraocular lens; UDVA, uncorrected distance visual acuity.

^a^ Comparison between continuous variables assessed by independent *t* test.

Subgroup analysis was performed based on the magnitude of preoperative astigmatism (Table 3). There were no differences between groups for patients with preoperative astigmatism less than 1.5 D. However, larger residual astigmatism was observed in the FSAK group compared with the TIOL group in the subgroup with preoperative astigmatism greater than 1.5 D at 3 months (mean [SD], 0.82 [0.66] D vs 0.53 [0.58] D; difference, 0.28 D; 95% CI, 0.07-0.50 D; *P* = .009). When stratified by astigmatism type, there were no differences between groups for WTR and oblique astigmatism during the follow-up visits (eTables 1 and 2 in Supplement 3). However, in the ATR subgroup, the refractive astigmatism was larger in the FSAK group than in the TIOL group at 3 months (mean [SD], 0.72 [0.62] D vs 0.50 [0.53] D; difference, 0.22 D; 95% CI, 0.01-0.44 D; *P* = .04).

**Table 3.** Primary and Key Secondary Outcomes at 3 Months in Different Preoperative Astigmatism Subgroups

Subgroups	FSAK	TIOL	Mean difference (95% CI)	*P* value^a^
Preoperative astigmatism <1.5 D, No. (%)	37 (38)	33 (34)		
Refractive astigmatism, mean (SD), D	0.36 (0.45)	0.55 (0.47)	−0.19 (−0.41 to 0.03)	.09
UDVA				
LogMAR, mean (SD)	0.08 (0.13)	0.17 (0.23)	−0.08 (−0.17 to 0.001)	.05
Snellen equivalent, mean	20/24	20/30		
CDVA				
LogMAR, mean (SD)	0.04 (0.10)	0.09 (0.15)	−0.04 (−0.10 to 0.01)	.13
Snellen equivalent, mean	20/22	20/25		
Keratometric astigmatism, mean (SD), D	0.74 (0.60)	1.32 (1.05)	−0.58 (−0.98 to −0.17)	.006
Preoperative astigmatism ≥1.5 D, No. (%)	61 (62)	65 (66)		
Refractive astigmatism, mean (SD), D	0.82 (0.66)	0.53 (0.58)	0.28 (0.07 to 0.50)	.009
UDVA				
LogMAR, mean (SD)	0.19 (0.22)	0.12 (0.15)	0.07 (0.004 to 0.14)	.04
Snellen equivalent, mean	20/31	20/26		
CDVA				
LogMAR, mean (SD)	0.09 (0.20)	0.06 (0.13)	0.03 (−0.03 to 0.09)	.27
Snellen equivalent, mean	20/25	20/23		
Keratometric astigmatism, mean (SD), D	0.99 (0.66)	1.49 (0.89)	−0.49 (−0.81 to −0.18)	.004

Abbreviations: CDVA, corrected distance visual acuity; D, diopter; FSAK, femtosecond laser arcuate keratotomy; TIOL, toric intraocular lens; UDVA, uncorrected distance visual acuity.

^a^ Comparison between continuous variables assessed by independent *t* test.

### Visual Acuity

For the secondary outcomes, both groups demonstrated significant improvements in visual acuity. There were no differences in UDVA or CDVA between the 2 groups at each follow-up visit (Table 2 and eTables 3 and 4 in Supplement 3). At 3 months postoperatively, the mean (SD) UDVA was 0.15 (0.20) logMAR (Snellen equivalent, 20/28) for the FSAK group and 0.14 (0.19) logMAR (Snellen equivalent, 20/28) for the TIOL group (difference, 0.01 D; 95% CI, −0.04 to 0.06 D; *P* = .71). The mean (SD) CDVA was 0.07 (0.16) logMAR (Snellen equivalent, 20/23) for the FSAK group and 0.07 (0.14) logMAR (Snellen equivalent, 20/23) for the TIOL group (difference, 0.004 D; 95% CI, −0.04 to 0.05 D; *P* = .82).

Subgroup analyses were performed based on the type and magnitude of preoperative astigmatism (Table 3 and eTables 2-4 in Supplement 3). For patients with preoperative astigmatism exceeding 1.5 D, mean (SD) UDVA was better in the TIOL group (0.12 [0.15] D logMAR; Snellen equivalent, 20/26) compared with the FSAK group (0.19 [0.22] D logMAR; Snellen equivalent, 20/31) at 3 months (difference, −0.07 D; 95% CI, −0.14 to −0.004 D; *P* = .04) (Table 3). No differences were found for CDVA between subgroups at the follow-up period (eTable 4 in Supplement 3).

### Keratometric Astigmatism

Postoperatively, a reduction of keratometric astigmatism was observed in the FSAK group compared with the TIOL group (Table 2 and eTable 5 in Supplement 3). At 3 months postoperatively, the mean (SD) keratometric astigmatism was 0.90 (0.64) D for the FSAK group and 1.43 (1.08) D for the TIOL group (difference, −0.53 D; 95% CI, −0.82 to −0.24 D; *P* = .002).

### Vector Analysis

Further astigmatic analysis was performed using the Alpins methods (eTable 6 in Supplement 3).^26,27^ At 3 months postoperatively, the mean (SD) surgically induced astigmatism (SIA) value was larger in the TIOL group compared with the FSAK group (1.58 [0.81] D vs 1.24 [0.72] D; difference, 0.34 D; 95% CI, 0.12-0.56 D; *P* = .002). The mean (SD) correction index, which represents the ratio of SIA to target induced astigmatism (TIA), was also larger in the TIOL group than the FSAK group (0.95 [0.39] vs 0.79 [0.29]; difference, 0.16; 95% CI, 0.06-0.26 D; *P* = .001) at 3 months postoperatively. eFigure 2 in Supplement 3 presents the scatterplot of TIA vs SIA and illustrates the distribution of angle of error 3 months after surgery between the 2 groups, indicating that more patients in the FSAK group were undercorrected.

### Adverse Events

In the FSAK group, a case of corneal perforation was observed at the temporal arcuate incision site due to eye movement during the incision separation (eFigure 3 in Supplement 3). Regarding TIOL, 1 case exhibited misalignment of over 10°, necessitating secondary repositioning surgery. No other adverse events occurred in either group.

## Discussion

This randomized clinical trial compared the refractive outcomes of FSAK and TIOL implantation in patients with mild to moderate astigmatism undergoing FLACS. Both procedures exhibited favorable clinical outcomes, but no differences were found for refractive astigmatism or visual acuity. Specifically, for study participants with mild to moderate astigmatism undergoing femtosecond laser-assisted cataract surgery, for the primary outcome, FSAK was not superior to TIOL implantation for astigmatism correction. Vector analysis of the refractive astigmatism showed that the TIOL group exhibited larger SIA and correction index than the FSAK group, suggesting greater effectiveness in astigmatism correction, but this was not the primary outcome. Notably, when treating preoperative astigmatism exceeding 1.5 D and ATR astigmatism, the TIOL group demonstrated smaller refractive astigmatism compared with the FSAK group, but again, this was not the primary outcome.

No differences were found for refractive astigmatism between groups during the follow-up period. These findings align with previous studies comparing FSAK and TIOL with conventional cataract surgery.^16,18,20^ Lin et al^16^ reported no difference of refractive astigmatism between FSAK (mean [SD], 0.98 [0.61] D) and TIOL (mean [SD], 0.92 [0.72] D) groups through 6 months. Additionally, no differences were observed for UDVA and CDVA in our study, which were comparable with previous results.^17,20^

To better understand the factors that might underlie the effects of astigmatism correction, subgroup analyses were conducted based on astigmatism type and the magnitude of preoperative astigmatism. When the preoperative astigmatism exceeded 1.5 D, the TIOL group exhibited smaller residual astigmatism and better UDVA compared with the FSAK group. These results aligned with the results of subgroup analyses performed by Noh et al^19^ using a smaller sample. Another study, which exclusively included patients with moderate astigmatism, also concluded that TIOL implantation outperformed FSAK.^17^ Regarding the direction of preoperative astigmatism, the TIOL group had smaller residual astigmatism than FSAK group when correcting ATR astigmatism at 3 months postoperatively. For ATR astigmatism, the online calculator automatically adjusted the arc length by adding 5°, potentially introducing greater variability.^23,29^ Additionally, Chan et al^30^ observed a trend of undercorrection over time in eyes with ATR astigmatism, which could be attributed to the shift of the anterior corneal surface from WTR to ATR astigmatism and the increase in posterior ATR astigmatism associated with aging. Therefore, these findings support considering the direction of preoperative astigmatism and the effect of natural aging when designing astigmatism correction procedures.

Vector analysis may provide a more comprehensive understanding of astigmatism treatment outcomes. SIA, which represents the actual change in astigmatism from surgery, was larger in the TIOL group than the FSAK group, aligning with previous studies.^17,19^ Moreover, the TIOL group demonstrated a higher correction index than the FSAK group. These differences may be attributed to the larger difference vector and magnitude of error in the FSAK group, which supports considering a need to adjust the nomogram. In addition, the extensive wound healing process associated with FSAK incisions may contribute to the decreased correction that was noted.

Numerous nomograms for FSAK have been reported, although no consensus has been reached on which is the most effective. In this study, we used a modified version of the Donnenfeld nomogram, implementing arcuate incisions with a diameter of 8.5 mm and a depth of 90%. The FSAK group exhibited a mean correction index of 0.79, which aligns with findings from other studies.^11,19,29,30,31^ Wendelstein et al^31^ used the Castrop nomogram, specifically designed for FSAK, and reported a mean correction index of 0.92. Their arcuate incisions were performed with a diameter of 8.5 mm and a depth of 80%.^31^ However, the SIA of the main corneal incision was not considered in their study.^31^ Another study used a modified nomogram for the femtosecond laser system, creating arcuate incisions with a depth of 85% and a diameter of 9.0 mm, and the correction index of the FSAK group was only 0.71.^19^ As the AK incisions were not separated, the effectiveness of astigmatism correction may have been reduced.^19^ Although several factors have been identified to affect the correction efficacy of FSAK, there is currently no established standard nomogram. Further studies seem warranted to optimize and refine the existing nomograms.

### Limitations

The study has some limitations. First, the absence of masking among patients and surgeon may introduce a source of bias. Second, the follow-up period was limited to 3 months, preventing an assessment of the long-term stability and efficacy of both procedures. Third, because the primary outcome did not reveal a difference between groups, the secondary outcomes and subgroup analyses should be interpreted as exploratory, necessitating confirmation in subsequent clinical trials. Fourth, all the surgical procedures were performed by a single surgeon, which limits the generalizability of the results. Fifth, visual acuity was not measured using the ETDRS (from the Early Treatment Diabetic Retinopathy Study) approach. Sixth, only 1 eye of the patient was included, which precluded analysis of patient satisfaction, spectacle independence, and binocular visual acuity, etc. Finally, the corneal biomechanical parameters could be further analyzed to enhance our understanding of the mechanisms underlying FSAK and improve the efficacy of current nomograms.

## Conclusions

In conclusion, this randomized clinical trial compared the clinical outcomes of FSAK and TIOL implantation among patients undergoing FLACS. In patients with mild to moderate astigmatism undergoing FLACS, FSAK was not superior to TIOL implantation for astigmatism correction. Both procedures demonstrated comparable clinical outcomes in terms of refractive astigmatism and visual acuity, whereas TIOL implantation showed greater effectiveness in astigmatism correction, although this was not the primary outcome. Additionally, when addressing astigmatism exceeding 1.5 D and ATR astigmatism, TIOL implantation demonstrated greater efficacy over FSAK, but again, this was not the primary outcome. This investigation supports the possibility that FSAK can be performed together with femtosecond laser procedures, and therefore, might be considered when addressing mild astigmatism. Nevertheless, further studies with extended follow-up periods are necessary to refine and improve the existing nomogram, as well as to elucidate the underlying mechanisms of astigmatism correction.

## Notes

Supplement 1.
Trial Protocol.

Supplement 2.
Statistical Analysis Plan.

Supplement 3.
eMethods.
**eTable 1.** Refractive Astigmatism Between Groups at Other Follow-up Periods **eTable 2.** Primary and Key Secondary Outcomes at 3 Months in Different Astigmatism Type Subgroups **eTable 3.** Uncorrected Distance Visual Acuity Between Groups at Other Follow-Up Periods **eTable 4.** Corrected Distance Visual Acuity Between Groups at Other Follow-Up Periods **eTable 5.** Keratometric Astigmatism Between Groups at Other Follow-Up Periods **eTable 6.** Vector Analysis of Refractive Astigmatism at 3 Months Between the FSAK and TIOL Groups **eFigure 1.** Double-Angle Plot of Vector Analysis of Preoperative and Postoperative Astigmatism in the FSAK and TIOL Groups **eFigure 2.** Scatterplot of TIA vs SIA and Astigmatism Angle of Error in the FSAK and TIOL Groups **eFigure 3.** A Case of Corneal Perforation and Anterior Synechia in the FSAK Group

Supplement 4.
Data Sharing Statement.
