# Supplementary material for: Femtosecond Laser Arcuate Keratotomy vs Toric Intraocular Lens Implantation in Cataract Surgery: A Randomized Clinical Trial
Source: JAMA Ophthalmol. 2025 Jan 23;143(3):199–206. doi: 10.1001/jamaophthalmol.2024.5887 (PMC11926645; doi:10.1001/jamaophthalmol.2024.5887)
Supplement: Supplement 1. — Trial Protocol. [file jamaophthalmol-e245887-s001.pdf]

Structured Protocol

Femtosecond Laser Arcuate Keratotomy versus Toric Intraocular Lens Implantation in Cataract Surgery: A Randomized Clinical Trial

Sponsor-investigator

Ke YAO, MD, Professor and Chief. Eye Center, The Second Affiliated Hospital, School of Medicine, Zhejiang University, Zhejiang Provincial Key Laboratory of Ophthalmology, Zhejiang Provincial Clinical Research Center for Eye Diseases, Zhejiang Provincial Engineering Institute on Eye Diseases, Hangzhou, Zhejiang, China. E-mail address: xlren@zju.edu.cn.

Yibo Yu, MD, Eye Center, The Second Affiliated Hospital, School of Medicine, Zhejiang University, Zhejiang Provincial Key Laboratory of Ophthalmology, Zhejiang Provincial Clinical Research Center for Eye Diseases, Zhejiang Provincial Engineering Institute on Eye Diseases, Hangzhou, Zhejiang, China. E-mail address: yuyibo@zju.edu.cn.

TABLE OF CONTENTS

LIST OF ABBREVIATIONS 4
1. TRIAL INFORMATION 5
1.1 Title of Project 5
1.2 Funding 5
1.3 Trial Summary 5
2. BACKGROUND 7
3. STUDY DESIGN 7
3.1 Overview 7
3.2 Setting 7
3.3 Scheme 7
4. PARTICIPANTS 8
4.1 Criteria for Inclusion 8
4.2 Criteria for Exclusion 8
4.3 Sample Size 9
5. RANDOMIZATION 9
6. STUDY PROCEDURE 9
6.1 Preoperative Measurement 11
6.2 Enrollment and Informed Consent 11
6.3 Surgical Procedures 11
7. OUTCOME MEASURES 12
7.1 Primary Outcome 12
7.2 Secondary Outcome 12
7.3 Data Collection and Management 13
8. ADVERSE EVENTS 13
9. DATA ANALYTIC PLAN 13
9.1 Primary Outcome 13
9.2 Secondary Outcome 13

9.3 Statistical Methods..14
10. ETHICAL CONSIDERATION 15
REFERENCES..15

LIST OF ABBREVIATIONS

- AE: angle of error
- ATR: against-the-rule
- CA: coefficient of adjustment
- CI: correction index
- CDVA: corrected distance visual acuity
- D: diopters
- DV: difference vector
- FI: flattening index
- FLACS: femtosecond laser-assisted cataract surgery
- FSAK: femtosecond laser arcuate keratotomy
- IOL: intraocular lens
- IOP: intraocular pressure
- IOS: index of success
- logMAR: the Logarithm of the Minimum Angle of Resolution
- ME: magnitude of error
- SAE: serious adverse events
- SIA: surgically-induced astigmatism
- SD: standard deviation
- TIA: target-induced astigmatism
- TIOL: Toric intraocular lens
- UDVA: uncorrected distance visual acuity
- WTR: with-the-rule

1. TRIAL INFORMATION

1.1 Title of Project

Femtosecond Laser Arcuate Keratotomy (FSAK) versus Toric Intraocular Lens (TIOL) Implantation in Cataract Surgery: A Randomized Clinical Trial

1.2 Funding

This study will be supported by the National Natural Science Foundation of China (Grant numbers 82201158, 82271063, and 82371036).

1.3 Trial Summary

**Objective**	To compare the clinical outcomes of FSAK and TIOL implantation for astigmatism correction in cataract surgery.
**Trial Setting**	The randomized controlled clinical trial is planned to be conducted between October 2021 and September 2023 at the Eye Center of the Second Affiliated Hospital, School of Medicine, Zhejiang University, China
**Participants**	
**Sample Size**	A sample size of 196 (98 in each group) is estimated with consideration of a dropout rate of 20%.
**Inclusion Criteria**	Cataract patients aged from 18 to 80 years with regular corneal astigmatism ranging from 0.75 to 3.00 D.
**Exclusion Criteria**	1) ocular surface abnormalities such as irregular corneal astigmatism, corneal scarring, keratoconus, and pterygium<br>2) history of ocular trauma or surgery<br>3) presence of coexisting ocular disorders such as glaucoma, retinal vascular occlusive disease, retinal detachment, diabetic retinopathy, and any optic nerve-related pathologies<br>4) poorly dilated pupils with a diameter less than 5.0 mm<br>5) concurrent severe systemic diseases<br>6) lack of cooperation.
**Interventions**	Eligible patients will be randomly allocated with a ratio of

	1:1 into 2 groups.<br>1) FSAK: to create symmetrical paired corneal arcuate keratotomies using the femtosecond laser.<br>2) TIOL implantation: to implant a Toric IOL (Tecnis Toric ZCT, Johnson & Johnson Vision, Santa Ana, CA, USA).
**Follow-up plan**	Each patient will undergo examinations at 1 day, 1 week, 1 month, and 3 months postoperatively. Uncorrected distance visual acuity (UDVA), corrected distance visual acuity (CDVA), subjective manifest refraction, and corneal topography assessments will be performed at each visit.
**Primary outcome**	The subjective refraction of FSAK and TIOL groups achieved at 3 months postoperatively.
**Secondary outcomes**	UDVA, CDVA, corneal topography assessments, intraoperative and postoperative complications.

2. BACKGROUND

Cataract surgery has undergone a paradigm shift to refractive surgery in this modern era. Nevertheless, residual astigmatism continues to be one of the major factors influencing patients' visual quality and satisfaction. According to statistics, approximately 42% of cataract patients exhibit preoperative astigmatism exceeding 1.0 D and 11% demonstrate astigmatism greater than 2.0 D. However, very few cataract patients have received any treatment to correct preoperative astigmatism, highlighting the significant burden associated with residual astigmatism.

Among various approaches, both Toric IOL implantation and FSAK are promising and effective solutions for addressing astigmatism and there has been much interest in comparing their efficacy and safety in cataract surgery. The purpose of this study is to compare these two procedures for astigmatism correction among patients receiving FLACS and evaluate the underlying factors that influence the correction efficacy.

3. STUDY DESIGN

3.1 Overview

This is a prospective randomized clinical trial comparing the clinical outcomes of Toric IOL implantation and FSAK among patients receiving FLACS. A total of 196 cataract patients will be recruited in the study. Randomization will be performed with a 1:1 allocation. Participants will be designed to receive FLACS with either FSAK or TIOL implantation and followed up for a total of 12 weeks from the date of randomization. Follow-up visits will be set at 1 day, 1 week, 1 month, and 3 months postoperatively.

3.2 Setting

This trial will be conducted at the Eye Center of the Second Affiliated Hospital, School of Medicine, Zhejiang University, China. To ensure the homogeneity of surgical procedures between groups, phacoemulsification will be performed by the same experienced surgeon (Ke Yao). Potentially eligible individuals will be recruited from outpatient clinics.

3.3 Scheme

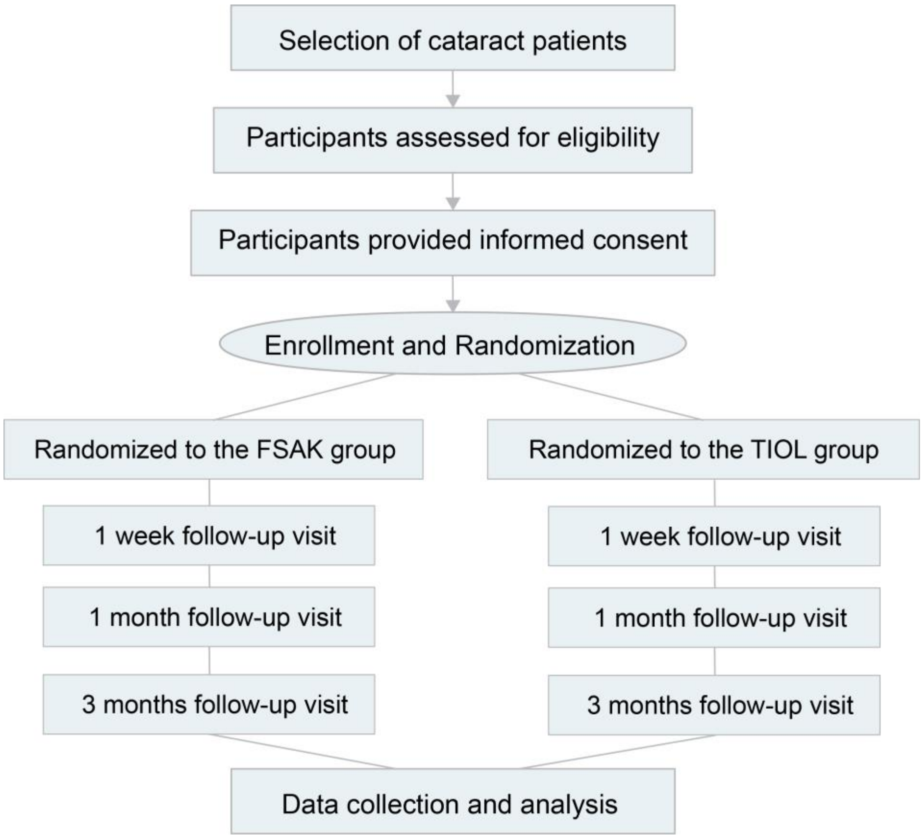

4. PARTICIPANTS

4.1 Criteria for Inclusion

- Patients with cataract schedule for phacoemulsification with posterior chamber IOL implantation
- Age between 18 to 80 years
- Regular corneal astigmatism ranging from 0.75 D to 3.00 D
- Pupil size of 6 mm or greater after dilation
- Have potential visual acuity of at least 20/25 Snellen (0.8 decimal) in the eye to be treated
- Voluntary participation and signed the informed consent

4.2 Criteria for Exclusion

- Ocular surface abnormalities such as irregular corneal astigmatism, corneal scarring, keratoconus, and pterygium
- History of ocular trauma or surgery
- Presence of coexisting ocular disorders such as glaucoma, retinal vascular occlusive disease, retinal detachment, diabetic retinopathy, and any optic

nerve-related pathologies

- Poorly dilated pupils with a diameter less than 5.0 mm
- Concurrent severe systemic diseases
- Lack of cooperation
- Concurrent participation or participation in any other clinical study within 30 days prior to the preoperative visit

4.3 Sample size

The sample size calculation was performed to determine the number of patients needed to detect a significant difference in postoperative refractive astigmatism between FSAK group and TIOL group. We considered a previous study where the mean postoperative refractive astigmatism was 0.780 D in the FSAK group and 0.834 D in the TIOL group, with a standard deviation of 0.106[3]. With a power of 90% and a significance level of 0.05, we used a two-sample t-test for the sample size calculation. Under these assumptions, the calculated sample size per group was 82 eyes. To account for potential loss to follow-up and non-compliance, we increased the sample size by 20%, resulting in a final target sample size of 98 eyes per group. The sample size was calculated using PASS 16.0 (NCSS, LLC, USA).

5. RANDOMIZATION

Eligible participants will be randomized to one of the following intervention groups: the FSAK group and the TIOL group. A computer-based random number table was generated with an allocation ratio of 1:1. Both the patients and surgeons were not masked to the interventions.

6. STUDY PROCEDURE

**Visit**	**Visit 1**	**Visit 2**	**Visit 3**	**Visit 4**	**Visit 5**	**Visit 6**
**Examination**	**Baseline**	**Operation**	**Postoperative 1 day**	**Postoperative 1 week**	**Postoperative 1 month**	**Postoperative 3 months**
Informed consent	√					
Eligibility	√					
FSAK or TIOL group		√				
UDVA, CDVA	√		√	√	√	√
Manifest refraction	√		√	√	√	√
Intraocular pressure	√		√	√	√	√
Slit lamp exam	√		√	√	√	√
Topography	√			√	√	√
Dry eye examination	√			√	√	√
Endothelial cell density	√			√	√	√
Contrast sensitivity						√
Adverse events			√	√	√	√

6.1 Preoperative Measurement

Each patient with age-related cataract and scheduled for FLACS with IOL implantation will be examined preoperatively. For each patient a detailed medical history will be recorded. The standard ophthalmic examination includes UDVA, CDVA, intraocular pressure (IOP) measurement, slit lamp examination, manifest refraction, keratometry or topography, cell density of cornea, IOL power calculation, fundus examination and optical coherence tomography. The cataract of the patient will be graded by slit lamp images by LOSC III grading system.

6.2 Enrollment and Informed Consent

After preoperative examination, the results will be assessed by the surgeon and the investigators for eligibility according to the inclusion and exclusion criteria. Eligible patients will be given a written information sheet in Chinese. Detailed contents will be explained by the investigators. If the patient would like to participate, the informed consent will be signed by the patient. Following the informed consent process, the patient may be scheduled for surgery.

6.3 Surgical Procedures

Surgical procedures will be in accordance with the routine practice. In this study, a single experienced surgeon from the Eye Center of the Second Affiliated Hospital, School of Medicine, Zhejiang University will perform cataract surgery. Prior to surgery, all the operative eye will undergo horizontal markings (0° and 180°) under the slit lamp with the patient seated upright to prevent cyclotorsion. Topical levofloxacin and pranoprofen will be administrated 4 times daily to the patients for 1 day before surgery. Pupillary dilation will be achieved using topical tropicamide every 10 minutes, 3 times preoperatively. Topical proparacaine hydrochloride will be administrated prior to femtosecond laser procedure. Nonpreserved adrenaline (1:10,000) will be injected intracamerally after the laser procedure if miosis developed.

All patients underwent femtosecond laser capsulotomy and nuclear fragmentation, followed by phacoemulsification and insertion of an IOL. After precise and centered docking of the suction ring (SoftFit Patient Interface; Alcon-LenSx, Inc.) onto the patient’s eye, the alignment of the procedural settings, including capsulotomy and

nuclear fragmentation, will be visualized with spectral domain optical coherence tomography using LenSx software (version 2.23, Alcon LenSx, Inc.). Capsulotomy with a diameter of 5.0 mm will be performed in all cases.

After the femtosecond laser pretreatment, a 2.0-mm single-plane primary corneal incision and a 0.8-mm side-port incision will be made manually using a keratome. The anterior capsule will be removed with capsule forceps, followed by phacoemulsification using a standard stop-and-chop technique. All procedural characteristics of the phacoemulsification will be consistent among the 2 groups.

In the FSAK group, manual alignment of the horizontal marks with the suction ring will be performed. Guided by spectral domain optical coherence tomography, symmetrical paired corneal arcuate keratotomies will be performed at an 8.5 mm diameter optical zone, with a depth of 90% corneal pachymetry. After standard phacoemulsification, a monofocal IOL (Tecnis ZCB00, Johnson & Johnson Vision, Santa Ana, CA, USA) will be implanted, and the arcuate incisions will be dissected with a blunt spatula to ensure complete separation.

In the TIOL group, the Toric IOL power and alignment axis will be calculated using the online calculator available at https://www.tecnistoriccalc.com. Following FLACS surgery, a Toric IOL (Tecnis Toric ZCT, Johnson & Johnson Vision, Santa Ana, CA, USA) will be implanted and adjusted to its final targeted position by aligning the Toric reference marks with the limbal axis marks.

Postoperatively, all patients will receive a standard postoperative regimen consisting of topical dexamethasone tobramycin 4 times per day for 2 weeks and pranoprofen 4 times per day for one month to the operated eye.

7. OUTCOME MEASURES

7.1 Primary Outcome

- The refractive astigmatism of FSAK and TIOL groups. The manifest refraction will be assessed at 1 day, 1 week, 1month, and 3 months postoperatively. The spherical equivalent and cylinder will be recorded and compared between groups.

7.2 Secondary Outcome

- UDVA
- CDVA

- Corneal topography assessments
- IOP
- Percentage of eyes that achieve refractive astigmatism at 1 month and 3 months postoperatively: within 0.25 D, 0.50 D, 0.75 D, 1.00D, 1.25 D, and 1.50 D.
- Toric IOL axis rotation at each postoperative visit: mean axis change, percentage of eyes with lens axis change within 5°, 10°, and 15°.
- Intraoperative and postoperative complications and adverse events
- Self-reported ocular symptoms

7.3 Data Collection and Management

All the original raw data and forms will be recorded by the investigators during the study. Each patient recruited will have a case report form to record all relevant clinical data in this clinical trial. The investigators will routinely generate reports and review effectiveness and safety data. To prevent missing data, patients will be encouraged at the time of informed consent to avoid missing study visits.

8. ADVERSE EVENTS

An adverse event is defined as any untoward medical occurrence, unintended disease or injury, or untoward clinical signs (including abnormal laboratory findings) in subjects, users or other persons, whether related to the study device. A serious adverse event (SAE) is defined as any untoward occurrence that is sight- or life-threatening, requires hospitalization, results in permanent impairment of body function, or necessitates medical or surgical intervention. All serious adverse events will be recorded using the SAE Case report form. Any SAE will be reported to the investigator and Institutional Ethics Committee. SAEs will be reported to the National Medical Products Administration within 24 hours.

The following list includes, but not limited to, SAEs that are anticipated and related to the study:

- Endophthalmitis or ocular infection
- Hypopyon
- Hyphema
- IOL dislocation
- Cystoid macular edema

- Pupillary block
- Retinal detachment/tear
- Acute corneal decompensation
- Corneal edema
- Chronic anterior uveitis that persists at 3 months postoperative
- Elevated IOP that persists at 3 months postoperative
- Toxic anterior segment syndrome
- Events requiring secondary surgical intervention (IOL removal or repositioning)

9. DATA ANALYTIC PLAN

In this study, the primary and secondary outcomes as defined above will be analyzed using the per-protocol principle. The final 3-month postoperative visit will be the key timeframe for all the outcomes. Data from other study visits may be reported for supportive analysis. All adverse events associated with the study intervention or occurred during the study duration will be addressed promptly and recorded.

9.1 Primary Outcome

The primary outcome of the study is refractive astigmatism. Astigmatic analysis will be performed using the Alpins method[4,5]. In brief, target-induced astigmatism (TIA) is defined as the intended astigmatic correction with magnitude and axis. Surgically-induced astigmatism (SIA) represents the actual change in astigmatism achieved by the surgery. Difference vector (DV) quantifies the induced astigmatic change necessary for the initial surgery to reach its intended target, ideally set at zero. Other parameters are calculated from these three vectors. The correction index (CI) indicated the ratio of SIA to TIA, with a value exceeding 1.0 suggesting overcorrection, and below 1.0 suggesting undercorrection. The coefficient of adjustment (CA) denoted the ratio of TIA to SIA. The magnitude of error (ME) represents the arithmetic difference between SIA and TIA, while the angle of error (AE) reflects the disparity in the axis between SIA and TIA. The index of success (IOS) is defined as the ratio of DV to TIA, and the flattening index (FI) is a measure of SIA’s effect on the astigmatic change along the intended axis.

9.2 Secondary Outcomes

For secondary outcomes, visual acuity values will be converted to LogMAR prior to analysis. Descriptive statistics for all eyes will be reported for mean LogMAR monocular uncorrected and best corrected distance visual acuity. In addition, the frequency and proportion of eyes that achieve refractive astigmatism at 1 month and 3 months postoperatively within 0.25 D, 0.50 D, 0.75 D, 1.00D, 1.25 D, and 1.50 D will be reported. The keratometric astigmatism, determined by corneal topography assessments, will be compared between groups.

9.3 Statistical Methods

Descriptive statistics may include mean, standard deviation, median, minimum, maximum for continuous data with frequency and proportion reported for categorical data. Variable normality will be assessed using the Shapiro-Wilk test. Wilcoxon Mann-Whitney U tests or Two-sample t-test will be used to comparing continuous variables between groups. For categorical variables, Pearson chi-square tests will be employed. All P values will be based on 2-sided tests ($P < 0.05$ considered statistically significant). Data analysis will be conducted using SPSS Statistics 26.0 (IBM, Armonk, NY, USA).

10. ETHICAL CONSIDERATION

The trial will be conducted in accordance with the ethical principles of the Declaration of Helsinki and Guideline for Good Clinical Practice in China. The investigator is responsible for notifying the Institutional Ethics Committee of reportable adverse events as well as any other circumstance in which additional procedures outside the protocol were conducted to eliminate hazards to participants. Ethical approval has been received from the ethics committee of Eye Center of the Second Affiliated Hospital of Zhejiang University (20210847).

REFERENCES

1. Keshav V, Henderson BA. Astigmatism Management with Intraocular Lens Surgery. Ophthalmology. 2021;128(11):e153-e163. doi:10.1016/j.ophtha.2020.08.011

2. Day AC, Dhariwal M, Keith MS, et al. Distribution of preoperative and postoperative astigmatism in a large population of patients undergoing cataract surgery in the UK. Br J Ophthalmol. 2019;103(7):993-1000.

doi:10.1136/bjophthalmol-2018-312025

3. Yoo A, Yun S, Kim JY, et al. Femtosecond Laser-assisted Arcuate Keratotomy Versus Toric IOL Implantation for Correcting Astigmatism. J Refract Surg. 2015;31:574–8.

4. Alpins N. Astigmatism analysis by the Alpins method. *J Cataract Refract Surg*. 2001;27(1):31-49. doi:10.1016/s0886-3350(00)00798-7

5. Alpins NA, Goggin M. Practical astigmatism analysis for refractive outcomes in cataract and refractive surgery. *Surv Ophthalmol*. 2004;49(1):109-122. doi:10.1016/j.survophthal.2003.10.010
