# Supplementary material for: Femtosecond Laser Arcuate Keratotomy vs Toric Intraocular Lens Implantation in Cataract Surgery: A Randomized Clinical Trial
Source: JAMA Ophthalmol. 2025 Jan 23;143(3):199–206. doi: 10.1001/jamaophthalmol.2024.5887 (PMC11926645; doi:10.1001/jamaophthalmol.2024.5887)
Supplement: Supplement 2. — Statistical Analysis Plan. [file jamaophthalmol-e245887-s002.pdf]

Statistical Analysis Plan

Femtosecond Laser Arcuate Keratotomy versus Toric Intraocular Lens Implantation in Cataract Surgery: A Randomized Clinical Trial

Sponsor-investigator

Ke YAO, MD, Professor and Chief. Eye Center, The Second Affiliated Hospital, School of Medicine, Zhejiang University, Zhejiang Provincial Key Laboratory of Ophthalmology, Zhejiang Provincial Clinical Research Center for Eye Diseases, Zhejiang Provincial Engineering Institute on Eye Diseases, Hangzhou, Zhejiang, China. E-mail address: xlren@zju.edu.cn.

Yibo Yu, MD, Eye Center, The Second Affiliated Hospital, School of Medicine, Zhejiang University, Zhejiang Provincial Key Laboratory of Ophthalmology, Zhejiang Provincial Clinical Research Center for Eye Diseases, Zhejiang Provincial Engineering Institute on Eye Diseases, Hangzhou, Zhejiang, China. E-mail address: yuyibo@zju.edu.cn.

The Statistical Analysis Plan provides details of the analyses to be performed for key study outcomes. The final 3-month postoperative follow-up visit will be the key timeframe for all the primary and secondary outcomes. Outcomes from other follow-up visits (1 day, 1 week, and 1 month postoperative) will be reported for additional analysis. Any complications or adverse events will be evaluated at all visits. Descriptive statistics may include mean, standard deviation, median, minimum, maximum for continuous data with frequency and proportion reported for categorical data. Variable normality will be assessed using the Shapiro-Wilk test. Wilcoxon Mann-Whitney U tests or Two-sample t-test will be used to comparing continuous variables between groups. For categorical variables, Pearson chi-square tests will be employed. All P values will be based on 2-sided tests ($P < 0.05$ considered statistically significant). Data analysis will be conducted using SPSS Statistics 26.0 (IBM, Armonk, NY, USA).

1. PRIMARY OUTCOME

The primary outcome of the study is refractive astigmatism. Postoperatively, each patient will undergo subjective refraction examination at each follow-up visit. The results of refractive astigmatism, both the magnitude and the axis, will be recorded and compared between groups. Descriptive statistics will be reported for mean refractive astigmatism, intended astigmatism, preoperative astigmatism, and spherical equivalent. In addition, the frequency and proportion of eyes within ± 0.50 D and ± 1.00 D will be reported.

Astigmatism, with its magnitude and axis, is best described mathematically by a vector. This allows combination of magnitude and direction to be expressed in a single mathematical expression. In our study, astigmatic analysis will be performed using the Alpins methods[1,2,3]. Prior to vector analysis, all the data will be transformed according to the Alpins methods. Several basic data variables and calculated vector quantities are defined. In brief, target-induced astigmatism (TIA) is defined as the intended astigmatic correction with magnitude and axis. Surgically-induced astigmatism (SIA) represents the actual change in astigmatism achieved by the surgery. Difference vector (DV) quantifies the induced astigmatic change necessary for the initial surgery to reach its intended target, ideally set at zero. Other parameters are calculated from these three vectors. The correction index (CI) is the ratio of SIA to

TIA, with a value exceeding 1.0 suggesting overcorrection, and below 1.0 suggesting undercorrection. The coefficient of adjustment (CA) denotes the ratio of TIA to SIA. The magnitude of error (ME) represents the arithmetic difference between SIA and TIA, while the angle of error (AE) reflects the disparity in the axis between SIA and TIA. The index of success (IOS) is defined as the ratio of DV to TIA, and the flattening index (FI) is a measure of SIA's effect on the astigmatic change along the intended axis.

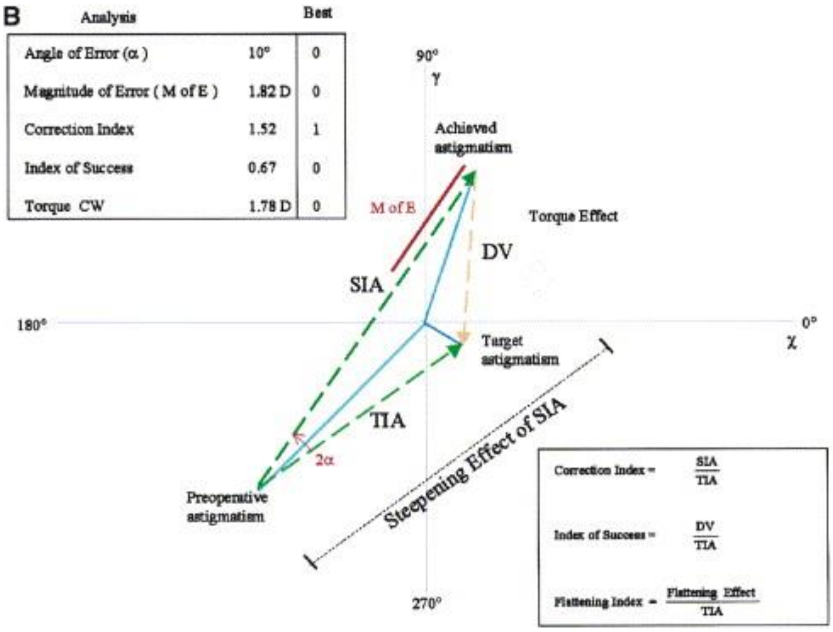

Figure 1. Illustration of vector analysis based on the Alpins methods[3]

2. SECONDARY OUTCOME

2.1 Visual Acuity

For visual acuity, the values will be converted to LogMAR prior to analysis. Descriptive statistics for all eyes will be reported for mean LogMAR uncorrected and best corrected distance visual acuity.

2.2 Corneal Endothelial Cell

Descriptive statistics will be reported for corneal endothelial cell data collected preoperatively and postoperatively.

2.3 Residual Refraction

The frequency and proportion of eyes that achieve refractive astigmatism at 1 month and 3 months postoperatively within 0.25 D, 0.50 D, 0.75 D, 1.00D, 1.25 D, and 1.50 D will be reported.

2.4 Toric IOL Rotational Stability

For Toric IOL implantation group, the axis change, defined as the absolute difference between IOL axis at operative visit and each follow-up visit, will be reported. Mean absolute rotation will also be reported for all visits using descriptive statistics.

2.5 Adverse Events

The frequency and proportion of eyes with any intraoperative and postoperative complications or adverse events will also be reported.

2.6 Self-reported ocular symptoms

The ocular or visual symptoms and questionnaire responses will be reported.

3. SAMPLE SIZE CALCULATIONS

The sample size calculation was performed to determine the number of patients needed to detect a significant difference in postoperative refractive astigmatism between FSAK group and TIOL group. We considered a previous study where the mean postoperative refractive astigmatism was 0.780 D in the FSAK group and 0.834 D in the TIOL group, with a standard deviation of 0.106[4]. With a power of 90% and a significance level of 0.05, we used a two-sample t-test for the sample size calculation. Under these assumptions, the calculated sample size per group was 82 eyes. To account for potential loss to follow-up and non-compliance, we increased the sample size by 20%, resulting in a final target sample size of 98 eyes per group. The sample size was calculated using PASS 16.0 (NCSS, LLC, USA).

References

1. Eydelman MB, Drum B, Holladay J, et al. Standardized analyses of correction of astigmatism by laser systems that reshape the cornea. J Refract Surg. 2006;22:81–95. doi: 10.3928/1081-597X-20060101-16

2. Alpins N. Astigmatism analysis by the Alpins method. *J Cataract Refract Surg.*

2001;27(1):31-49. doi:10.1016/s0886-3350(00)00798-7

3. Alpins NA, Goggin M. Practical astigmatism analysis for refractive outcomes in cataract and refractive surgery. *Surv Ophthalmol*. 2004;49(1):109-122. doi:10.1016/j.survophthal.2003.10.010

4. Yoo A, Yun S, Kim JY, Kim MJ, Tchah H. Femtosecond Laser-assisted Arcuate Keratotomy Versus Toric IOL Implantation for Correcting Astigmatism. J Refract Surg. 2015;31(9):574-578. doi:10.3928/1081597X-20150820-01
