# Supplementary material for: Femtosecond Laser Arcuate Keratotomy vs Toric Intraocular Lens Implantation in Cataract Surgery: A Randomized Clinical Trial
Source: JAMA Ophthalmol. 2025 Jan 23;143(3):199–206. doi: 10.1001/jamaophthalmol.2024.5887 (PMC11926645; doi:10.1001/jamaophthalmol.2024.5887)
Supplement: Supplement 3. — eMethods. eTable 1. Refractive Astigmatism Between Groups at Other Follow-up Periods eTable 2. Primary and Key Secondary Outcomes at 3 Months in Different Astigmatism Type Subgroups eTable 3. Uncorrected Distance Visual Acuity Between Groups at Other Follow-Up Periods eTable 4. Corrected Distance Visual Acuity Between Groups at Other Follow-Up Periods eTable 5. Keratometric Astigmatism Between Groups at Other Follow-Up Periods eTable 6. Vector Analysis of Refractive Astigmatism at 3 Months Between the FSAK and TIOL Groups eFigure 1. Double-Angle Plot of Vector Analysis of Preoperative and Postoperative Astigmatism in the FSAK and TIOL Groups eFigure 2. Scatterplot of TIA vs SIA and Astigmatism Angle of Error in the FSAK and TIOL Groups eFigure 3. A Case of Corneal Perforation and Anterior Synechia in the FSAK Group [file jamaophthalmol-e245887-s003.pdf]

Supplementary Online Content

Zhong Y, Chen S, Wang H, et al. Femtosecond laser arcuate keratotomy vs toric intraocular lens implantation in cataract surgery: a randomized clinical trial. *JAMA Ophthalmol*. Published online January 23, 2025.
doi:10.1001/jamaophthalmol.2024.5887

eMethods.

eTable 1. Refractive Astigmatism Between Groups at Other Follow-up Periods

eTable 2. Primary and Key Secondary Outcomes at 3 Months in Different Astigmatism Type Subgroups

eTable 3. Uncorrected Distance Visual Acuity Between Groups at Other Follow-Up Periods

eTable 4. Corrected Distance Visual Acuity Between Groups at Other Follow-Up Periods

eTable 5. Keratometric Astigmatism Between Groups at Other Follow-Up Periods

eTable 6. Vector Analysis of Refractive Astigmatism at 3 Months Between the FSAK and TIOL Groups

eFigure 1. Double-Angle Plot of Vector Analysis of Preoperative and Postoperative Astigmatism in the FSAK and TIOL Groups

eFigure 2. Scatterplot of TIA vs SIA and Astigmatism Angle of Error in the FSAK and TIOL Groups

eFigure 3. A Case of Corneal Perforation and Anterior Synechia in the FSAK Group

This supplementary material has been provided by the authors to give readers additional information about their work.

© 2025 Zhong Y et al. *JAMA Neurology*.

eMethods.

Surgical Procedures

Surgical procedures were in accordance with the routine practice. In this study, a single experienced surgeon from the Eye Center of the Second Affiliated Hospital, School of Medicine, Zhejiang University performed all the cataract surgery. Prior to surgery, all the operative eye underwent horizontal markings (0° and 180°) under the slit lamp with the patient seated upright to prevent cyclotorsion. Topical levofloxacin and pranoprofen were administrated 4 times daily to the patients for 1 day before surgery. Pupillary dilation was achieved using topical tropicamide every 10 minutes, 3 times preoperatively. Topical proparacaine hydrochloride was administrated prior to femtosecond laser procedure. Nonpreserved adrenaline (1:10,000) was injected intracamerally after the laser procedure if miosis developed.

All patients underwent femtosecond laser capsulotomy and nuclear fragmentation, followed by phacoemulsification and insertion of an IOL. After precise and centered docking of the suction ring (SoftFit Patient Interface; Alcon-LenSx, Inc.) onto the patient's eye, the alignment of the procedural settings, including capsulotomy and nuclear fragmentation, were visualized with spectral domain optical coherence tomography using LenSx software (version 2.23, Alcon LenSx, Inc.). Capsulotomy with a diameter of 5.0 mm was performed in all cases.

After the femtosecond laser pretreatment, a 2.0-mm single-plane primary corneal incision and a 0.8-mm side-port incision were made manually using a keratome. The anterior capsule was removed with capsule forceps, followed by phacoemulsification using a standard stop-and-chop technique. All procedural characteristics of the phacoemulsification were consistent among the 2 groups.

In the FSAK group, manual alignment of the horizontal marks with the suction ring were performed. Guided by spectral domain optical coherence tomography, symmetrical paired corneal arcuate keratotomies were performed at an 8.5 mm diameter optical zone, with a depth of 90% corneal pachymetry. The arc length and position of the arcuate incisions were determined by the Donnenfeld nomogram, using the online calculator available at https://www.lricalculator.com[1]. Considering that the diameter of the arcuate incisions was 11.0 mm, geometric modification was applied to adjust the arc length, resulting in a corrected arc length of 8.5/11 of the original value. After standard phacoemulsification, a monofocal IOL

(Tecnis ZCB00, Johnson & Johnson Vision, Santa Ana, CA, USA) was implanted, and the arcuate incisions were dissected with a blunt spatula to ensure complete separation.

In the TIOL group, the Toric IOL power and alignment axis was calculated using the online calculator available at https://www.tecnistoriccalc.com[2]. Intraoperatively, the intended implantation axis was marked on the limbus by aligning a Mendez ring with the horizontal marks. Following FLACS surgery, a Toric IOL (Tecnis Toric ZCT, Johnson & Johnson Vision, Santa Ana, CA, USA) was implanted and adjusted to its final targeted position by aligning the Toric reference marks with the limbal axis marks.

Postoperatively, all patients received a standard postoperative regimen consisting of topical dexamethasone tobramycin 4 times per day for 2 weeks and pranoprofen 4 times per day for one month to the operated eye.

Statistical Analysis

Multiple Imputation

All analyses were performed on an intention-to-treat (ITT) basis. The ITT analysis set consisted of all patients who were randomized. Missing data were imputed using the multiple imputation technique, where continuous variables were imputed using predictive mean matching and categorical variables were imputed using a logistic regression model. Five complete datasets were generated, and analyses were performed on each of the five datasets. The results were pooled across imputations according to Rubin's rules[3]. All the data analyses were conducted using SPSS Statistics 26.0 (IBM, Armonk, NY, USA) and R version 3.6.2 (R Foundation for Statistical Computing, Vienna, Austria).

Post-hoc Subgroup Analyses

At the end of the trial but prior to analysis, we identified post-hoc subgroup analyses based on the baseline characteristics for primary and key secondary outcomes. In reference to a previous study, the subgroups were identified based on the magnitude of preoperative astigmatism and the type of astigmatism, respectively[4]. The primary and key secondary outcomes were analyzed within subgroups using the independent Student's t-tests.

Regarding the magnitude of preoperative astigmatism, we set the cutoff points at 1.5 D in reference to a previous study[4]. Regarding the type of astigmatism, subgroups of with-the-rule, against-the-rule and oblique astigmatism were chosen. By definition, with-the-rule

© 2025 Zhong Y et al. *JAMA Neurology*.

astigmatism is defined as steep corneal meridians at at 60° to 120°. Against-the-rule (ATR) astigmatism is defined as steep corneal meridians at either 0 to 30° or 150° to 180°. Oblique astigmatism is defined as all values in between. Both FSAK and TIOL groups exhibited similar distribution of each subgroup.

Vector Analysis

Astigmatism, with its magnitude and axis, is best described mathematically by a vector[5]. This allows combination of magnitude and direction to be expressed in a single mathematical expression. In our study, astigmatic analysis will be performed using the Alpins methods[5-7]. Prior to vector analysis, all the data will be transformed according to the Alpins methods. Several basic data variables and calculated vector quantities are defined. In brief, target-induced astigmatism (TIA) is defined as the intended astigmatic correction with magnitude and axis. Surgically-induced astigmatism (SIA) represents the actual change in astigmatism achieved by the surgery. Difference vector (DV) quantifies the induced astigmatic change necessary for the initial surgery to reach its intended target, ideally set at zero. Other parameters are calculated from these three vectors. The correction index (CI) is the ratio of SIA to TIA, with a value exceeding 1.0 suggesting overcorrection, and below 1.0 suggesting undercorrection. The coefficient of adjustment (CA) denotes the ratio of TIA to SIA. The magnitude of error (ME) represents the arithmetic difference between SIA and TIA, while the angle of error (AE) reflects the disparity in the axis between SIA and TIA. The index of success (IOS) is defined as the ratio of DV to TIA, and the flattening index (FI) is a measure of SIA's effect on the astigmatic change along the intended axis.

© 2025 Zhong Y et al. *JAMA Neurology*.

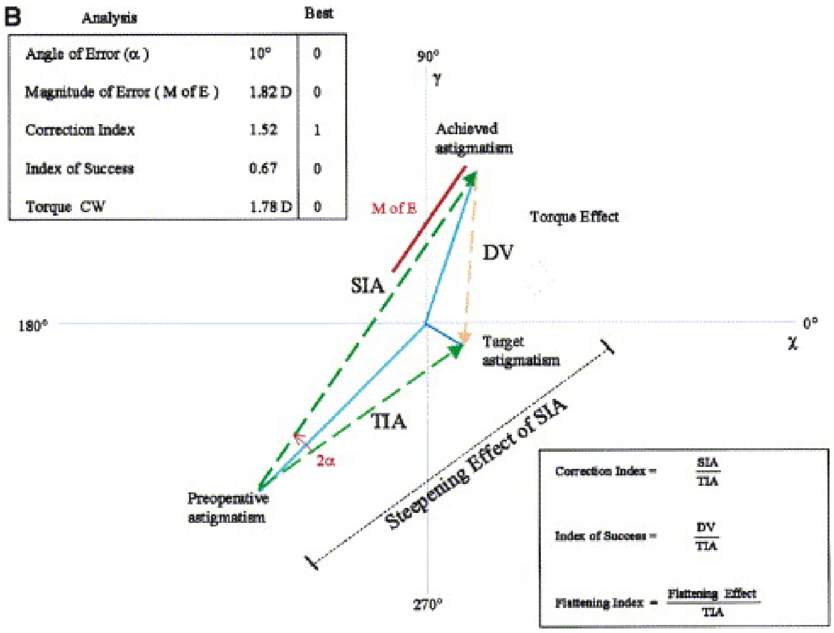

Figure 1. Illustration of vector analysis based on the Alpins methods[7]

References

1. Johnson & Johnson Surgical Vision, Inc. LRI Calculator. Welcome to the AMO LRI calculator software. Available at: https://www.lricalculator.com. Accessed November 20, 2023

2. Johnson & Johnson Surgical Vision, Inc. Calculator · Toric Calculator. Available at: https://www.tecnistoriccalc.com. Accessed November 26, 2023

3. Sterne JA, White IR, Carlin JB, Spratt M, Royston P, Kenward MG, Wood AM, Carpenter JR. Multiple imputation for missing data in epidemiological and clinical research: potential and pitfalls. BMJ. 2009;338:b2393

4. Noh H, Yoo Y-S, Shin KY, et al. Comparison of penetrating femtosecond laser-assisted astigmatic keratotomy and toric intraocular lens implantation for correction of astigmatism in cataract surgery. Sci Rep 2021;11:7340.

5. Eydelman MB, Drum B, Holladay J, et al. Standardized analyses of correction of astigmatism by laser systems that reshape the cornea. J Refract Surg. 2006;22:81–95. doi: 10.3928/1081-597X-20060101-16

6. Alpins N. Astigmatism analysis by the Alpins method. J Cataract Refract Surg. 2001;27(1):31-49. doi:10.1016/s0886-3350(00)00798-7

© 2025 Zhong Y et al. *JAMA Neurology*.

7. Alpins NA, Goggin M. Practical astigmatism analysis for refractive outcomes in cataract and refractive surgery. Surv Ophthalmol. 2004;49(1):109-122. doi:10.1016/j.survophthal.2003.10.010

© 2025 Zhong Y et al. *JAMA Neurology*.

eTable 1. Refractive Astigmatism Between Groups at Other Follow-up Periods

Period	FSAK	TIOL	Mean difference (95% CI)	*P* value[a]
**Total, No. (%)**	98 (100%)	98 (100%)		
1 day	0.77	0.59	0.18 (−0.03 to 0.39)	.10
1 week	0.69	0.56	0.14 (−0.04 to 0.32)	.14
1 month	0.67	0.55	0.12 (−0.04 to 0.28)	.13
**Preoperative astigmatism < 1.5 D, No. (%)**	37 (38%)	33 (34%)		
1 day	0.63	0.57	0.06 (−0.27 to 0.39)	.71
1 week	0.43	0.49	−0.06 (−0.27 to 0.16)	.60
1 month	0.41	0.55	−0.14 (−0.34 to 0.07)	.20
**Preoperative astigmatism ≥ 1.5 D, No. (%)**	61 (62%)	65 (66%)		
1 day	0.86	0.60	0.26 (−0.02 to 0.54)	.07
1 week	0.86	0.60	0.26 (0.01 to 0.51)	.040
1 month	0.83	0.55	0.28 (0.08 to 0.48)	.007
**With-the-rule astigmatism, No. (%)**	29 (30%)	30 (31%)		
1 day	0.64	0.57	0.06 (−0.29 to 0.41)	.73
1 week	0.40	0.47	−0.07 (−0.35 to 0.22)	.65
1 month	0.54	0.54	−0.01 (−0.27 to 0.26)	.97
**Against-the-rule astigmatism, No. (%)**	56 (57%)	54 (55%)		

© 2025 Zhong Y et al. *JAMA Neurology*.

1 day	0.86	0.56	0.30 (0.03 to 0.57)	.032
1 week	0.85	0.64	0.21 (−0.03 to 0.46)	.09
1 month	0.78	0.54	0.23 (0.03 to 0.44)	.026
**Oblique astigmatism, No. (%)**	13 (13%)	14 (14%)		
1 day	0.73	0.76	−0.02 (−0.77 to 0.72)	.95
1 week	0.68	0.45	0.23 (−0.21 to 0.67)	.31
1 month	0.53	0.60	−0.07 (−0.54 to 0.41)	.78

Abbreviations: FSAK, femtosecond laser arcuate keratotomy; TIOL, Toric intraocular lens; CI, confidence interval; D, diopters.

[a] Comparison between continuous variables assessed by independent Student's t-test.

.

© 2025 Zhong Y et al. *JAMA Neurology*.

eTable 2. Primary and Key Secondary Outcomes at 3 months in Different Astigmatism Type Subgroups

Subgroups	FSAK	TIOL	Mean difference (95% CI)	*P* value[a]
**With-the-rule astigmatism, No. (%)**	29 (30%)	30 (31%)		
Refractive astigmatism, mean, D	0.53	0.50	0.03 (−0.25 to 0.31)	.83
UDVA				
LogMAR, mean	0.10	0.11	−0.02 (−0.09 to 0.06)	.66
Snellen equivalent, mean	20/25	20/25		
CDVA				
LogMAR, mean	0.03	0.04	−0.02 (−0.06 to 0.03)	.54
Snellen equivalent, mean	20/20	20/20		
Keratometric astigmatism, mean, D	0.96	1.68	−0.72 (−1.16 to −0.28)	.001
**Against-the-rule astigmatism, No. (%)**	56 (57%)	54 (55%)		
Refractive astigmatism, mean, D	0.72	0.50	0.22 (0.01 to 0.44)	.041
UDVA				
LogMAR, mean	0.16	0.15	0.01 (−0.07 to 0.10)	.72
Snellen equivalent, mean	20/30	20/30		
CDVA				
LogMAR, mean	0.09	0.08	0.01 (−0.05 to 0.08)	.66
Snellen equivalent, mean	20/25	20/25		
Keratometric astigmatism, mean, D	0.84	1.33	−0.49 (−0.82 to −0.17)	.006
**Oblique astigmatism, No. (%)**	13 (13%)	14 (14%)		

© 2025 Zhong Y et al. *JAMA Neurology*.

Refractive astigmatism, mean, D	0.50	0.48	0.02 (−0.50 to 0.53)	.95
UDVA				
LogMAR, mean	0.19	0.14	0.05 (−0.10 to −0.20)	.51
Snellen equivalent, mean	20/30	20/25		
CDVA				
LogMAR, mean	0.10	0.09	0.01 (−0.11 to 0.12)	.89
Snellen equivalent, mean	20/25	20/25		
Keratometric astigmatism, mean, D	1.00	1.27	−0.27 (−0.82 to 0.27)	.32

Abbreviations: FSAK, femtosecond laser arcuate keratotomy; TIOL, Toric intraocular lens; CI, confidence interval; D, diopters; UDVA, uncorrected distance visual acuity; CDVA, corrected distance visual acuity; LogMAR, logarithm of the minimum angle of resolution.

[a] Comparison between continuous variables assessed by independent Student's t-test.

.

© 2025 Zhong Y et al. *JAMA Neurology*.

eTable 3. Uncorrected Distance Visual Acuity Between Groups at Other Follow-up Periods

Period	FSAK		TIOL		Mean difference (95% CI)	*P* value[a]
	LogMAR, mean	Snellen equivalent, mean	LogMAR, mean	Snellen equivalent, mean		
**Total, No. (%)**	98 (100%)		98 (100%)			
1 day	0.21	20/32	0.19	20/31	0.02 (−0.04 to 0.08)	.56
1 week	0.17	20/30	0.13	20/27	0.04 (−0.01 to 0.09)	.15
1 month	0.15	20/28	0.14	20/28	0.01 (−0.04 to 0.06)	.66
**Preoperative astigmatism < 1.5 D, No. (%)**	37 (38%)		33 (34%)			
1 day	0.14	20/28	0.22	20/33	−0.08 (−0.17 to 0.02)	.11
1 week	0.13	20/27	0.15	20/28	−0.02 (−0.11 to 0.07)	.62
1 month	0.11	20/26	0.18	20/30	−0.07 (−0.16 to 0.02)	.13
**Preoperative astigmatism ≥ 1.5 D, No. (%)**	61 (62%)		65 (66%)			
1 day	0.25	20/36	0.17	20/30	0.07 (−0.004 to 0.15)	.06
1 week	0.20	20/32	0.13	20/27	0.08 (0.006 to 0.15)	.032
1 month	0.17	20/30	0.12	20/26	0.06 (−0.003 to 0.12)	.06
**With-the-rule astigmatism, No. (%)**	29 (30%)		30 (31%)			
1 day	0.14	20/28	0.13	20/27	0.01 (−0.07 to 0.09)	.84
1 week	0.10	20/25	0.10	20/25	0.002 (−0.07 to 0.07)	.95

© 2025 Zhong Y et al. *JAMA Neurology*.

1 month	0.11	20/26	0.13	20/27	−0.02 (−0.10 to 0.06)	.62
**Against-the-rule astigmatism, No. (%)**	56 (57%)		54 (55%)			
1 day	0.23	20/34	0.21	20/32	0.02 (−0.06 to 0.11)	.62
1 week	0.20	20/32	0.15	20/28	0.05 (−0.03 to 0.13)	.25
1 month	0.16	20/29	0.14	20/28	0.02 (−0.05 to 0.09)	.63
**Oblique astigmatism, No. (%)**	13 (13%)		14 (14%)			
1 day	0.26	20/36	0.23	20/34	0.02 (−0.17 to 0.22)	.80
1 week	0.21	20/32	0.13	20/27	0.07 (−0.05 to 0.22)	.24
1 month	0.19	20/31	0.14	20/28	0.05 (−0.08 to 0.18)	.45

Abbreviations: FSAK, femtosecond laser arcuate keratotomy; TIOL, Toric intraocular lens; LogMAR, logarithm of the minimum angle of resolution; CI, confidence interval; D, diopters.

[a] Comparison between continuous variables assessed by independent Student's t-test.

© 2025 Zhong Y et al. *JAMA Neurology*.

eTable 4. Corrected Distance Visual Acuity Between Groups at Other Follow-up Periods

Period	FSAK		TIOL		Mean difference (95% CI)	*P* value[a]
	LogMAR, mean	Snellen equivalent, mean	LogMAR, mean	Snellen equivalent, mean		
**Total, No. (%)**	98 (100%)		98 (100%)			
1 day	0.12	20/26	0.11	20/26	0.01 (−0.04 to 0.06)	.67
1 week	0.09	20/25	0.07	20/23	0.03 (−0.02 to 0.07)	.26
1 month	0.08	20/24	0.07	20/23	0.01 (−0.03 to 0.05)	.54
**Preoperative astigmatism < 1.5 D, No. (%)**	37 (38%)		33 (34%)			
1 day	0.07	20/23	0.13	20/27	−0.06 (−0.13 to 0.01)	.11
1 week	0.08	20/24	0.09	20/25	−0.01 (−0.09 to 0.06)	.75
1 month	0.06	20/23	0.09	20/25	−0.03 (−0.09 to 0.03)	.32
**Preoperative astigmatism ≥ 1.5 D, No. (%)**	61 (62%)		65 (66%)			
1 day	0.15	20/28	0.10	20/25	0.05 (−0.01 to 0.11)	.12
1 week	0.10	20/25	0.06	20/23	0.05 (−0.01 to 0.10)	.10
1 month	0.09	20/25	0.05	20/22	0.04 (−0.02 to 0.09)	.18
**With-the-rule astigmatism, No. (%)**	29 (30%)		30 (31%)			
1 day	0.06	20/23	0.06	20/23	−0.01 (−0.07 to 0.06)	.87
1 week	0.04	20/22	0.04	20/22	−0.002 (−0.06 to 0.06)	.94
1 month	0.04	20/22	0.05	20/22	−0.02 (−0.07 to 0.04)	.67

© 2025 Zhong Y et al. *JAMA Neurology*.

**Against-the-rule astigmatism, No. (%)**	56 (57%)		54 (55%)			
1 day	0.14	20/28	0.13	20/27	0.02 (−0.05 to 0.09)	.65
1 week	0.12	20/26	0.08	20/24	0.04 (−0.03 to 0.10)	.31
1 month	0.09	20/25	0.07	20/23	0.02 (−0.04 to 0.08)	.51
**Oblique astigmatism, No. (%)**	13 (13%)		14 (14%)			
1 day	0.15	20/28	0.13	20/27	0.02 (−0.10 to 0.14)	.78
1 week	0.11	20/26	0.07	20/23	0.04 (−0.05 to 0.13)	.37
1 month	0.11	20/26	0.08	20/24	0.03 (−0.07 to 0.13)	.53

Abbreviations: FSAK, femtosecond laser arcuate keratotomy; TIOL, Toric intraocular lens; LogMAR, logarithm of the minimum angle of resolution; CI, confidence interval; D, diopters.

[a] Comparison between continuous variables assessed by independent Student's t-test.

© 2025 Zhong Y et al. *JAMA Neurology*.

eTable 5. Keratometric Astigmatism Between Groups at Other Follow-up Periods

Period	FSAK	TIOL	Mean difference (95% CI)	*P* value[a]
**Total, No. (%)**	98 (100%)	98 (100%)		
1 week	1.25	1.70	−0.45 (−0.72 to −0.19)	<.001
1 month	1.00	1.60	−0.60 (−0.83 to −0.39)	<.001
**Preoperative astigmatism < 1.5 D, No. (%)**	37 (38%)	33 (34%)		
1 week	1.06	1.42	−0.36 (−0.77 to 0.04)	.08
1 month	0.88	1.49	−0.61 (−1.06 to −0.16)	.011
**Preoperative astigmatism ≥ 1.5 D, No. (%)**	61 (62%)	65 (66%)		
1 week	1.36	1.85	−0.48 (−0.84 to −0.13)	.007
1 month	1.07	1.67	−0.60 (−0.83 to −0.37)	<.001
**With-the-rule astigmatism, No. (%)**	29 (30%)	30 (31%)		
1 week	1.01	1.86	−0.85 (−1.34 to −0.36)	<.001
1 month	0.90	1.72	−0.82 (−1.17 to −0.47)	<.001
**Against-the-rule astigmatism, No. (%)**	56 (57%)	54 (55%)		
1 week	1.24	1.72	−0.47 (−0.78 to −0.17)	.002
1 month	1.00	1.60	−0.61 (−0.86 to −0.36)	<.001
**Oblique astigmatism, No. (%)**	13 (13%)	14 (14%)		
1 week	1.77	1.32	0.46 (−0.48 to 1.39)	.34
1 month	1.20	1.37	−0.17 (−0.78 to 0.44)	.57

Abbreviations: FSAK, femtosecond laser arcuate keratotomy; TIOL, Toric intraocular lens; CI, confidence interval; D, diopters.

[a] Comparison between continuous variables assessed by independent Student's t-test.

© 2025 Zhong Y et al. *JAMA Neurology.*

eTable 6. Vector analysis of refractive astigmatism at 3 months between the FSAK and TIOL groups

Parameters	FSAK (n=98)	TIOL (n=98)	Mean difference (95% CI)	*P*[a]
Target induced astigmatism	1.56	1.67	−0.11 (−0.32 to 0.10)	.32
Surgically induced astigmatism	1.24	1.58	−0.34 (−0.56 to −0.12)	.002
Difference vector	0.64	0.54	0.11 (−0.06 to 0.27)	.21
Magnitude of error	0.41	0.29	0.12 (0.002 to 0.23)	.046
Angle of error	12.50	10.22	2.27 (−2.48 to 7.03)	.35
Correction index	0.79	0.95	−0.16 (−0.26 to −0.06)	.001
Coefficient of adjustment	1.60	1.34	0.26 (−0.15 to 0.67)	.21
Flattening index	0.63	0.73	−0.10 (−0.20 to 0.01)	.06
Index of success	0.41	0.39	0.02 (−0.10 to 0.14)	.77

Abbreviations: FSAK, femtosecond laser arcuate keratotomy; TIOL, Toric intraocular lens; CI, confidence interval.

[a] Comparison between continuous variables assessed by independent Student's t-test.

© 2025 Zhong Y et al. *JAMA Neurology*.

eFigure 1. Double-angle plot of vector analysis of preoperative and postoperative astigmatism in the FSAK and TIOL groups.

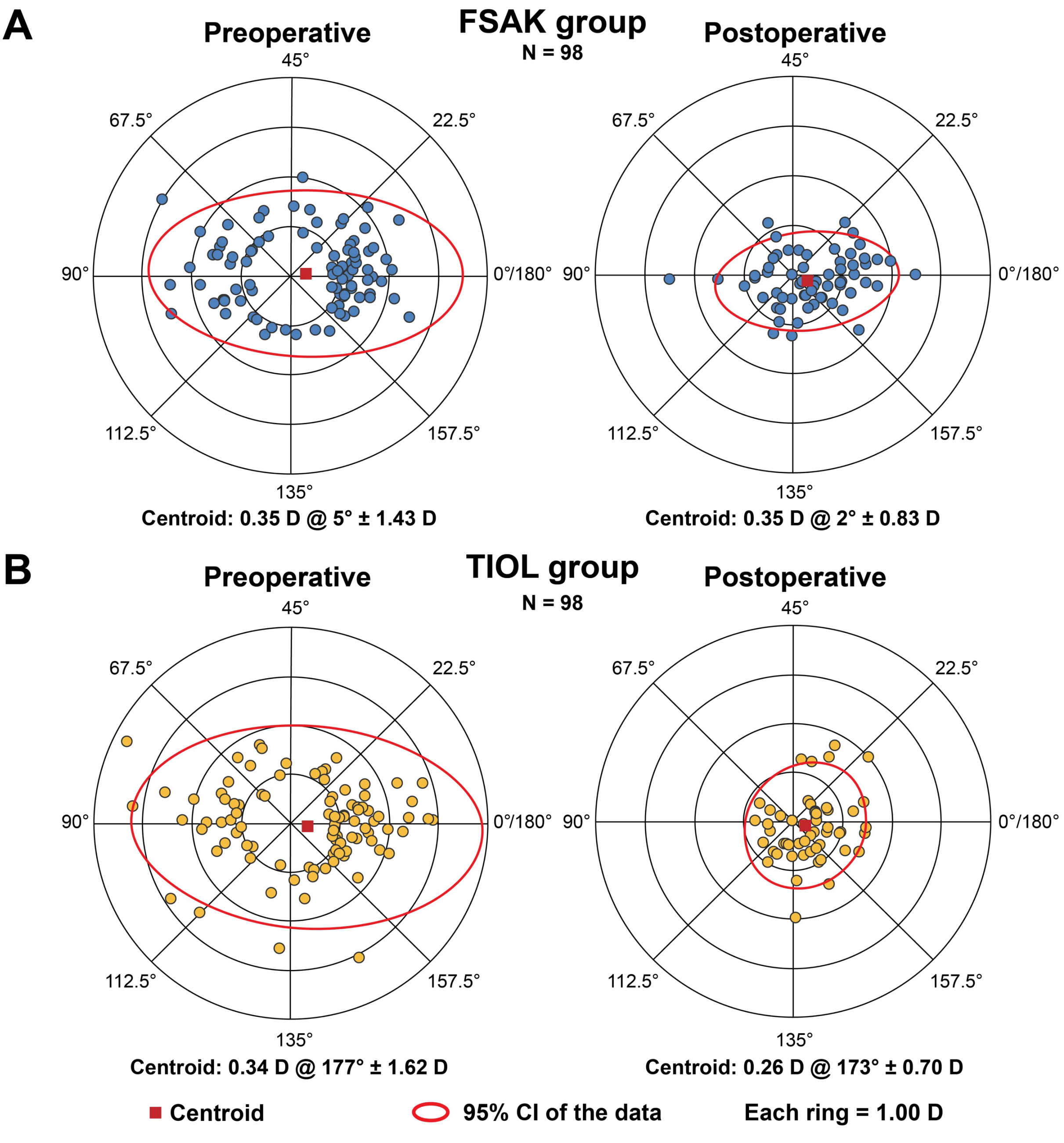

© 2025 Zhong Y et al. *JAMA Neurology*.

eFigure 2. Scatterplot of TIA versus SIA and astigmatism angle of error in the FSAK and TIOL groups.

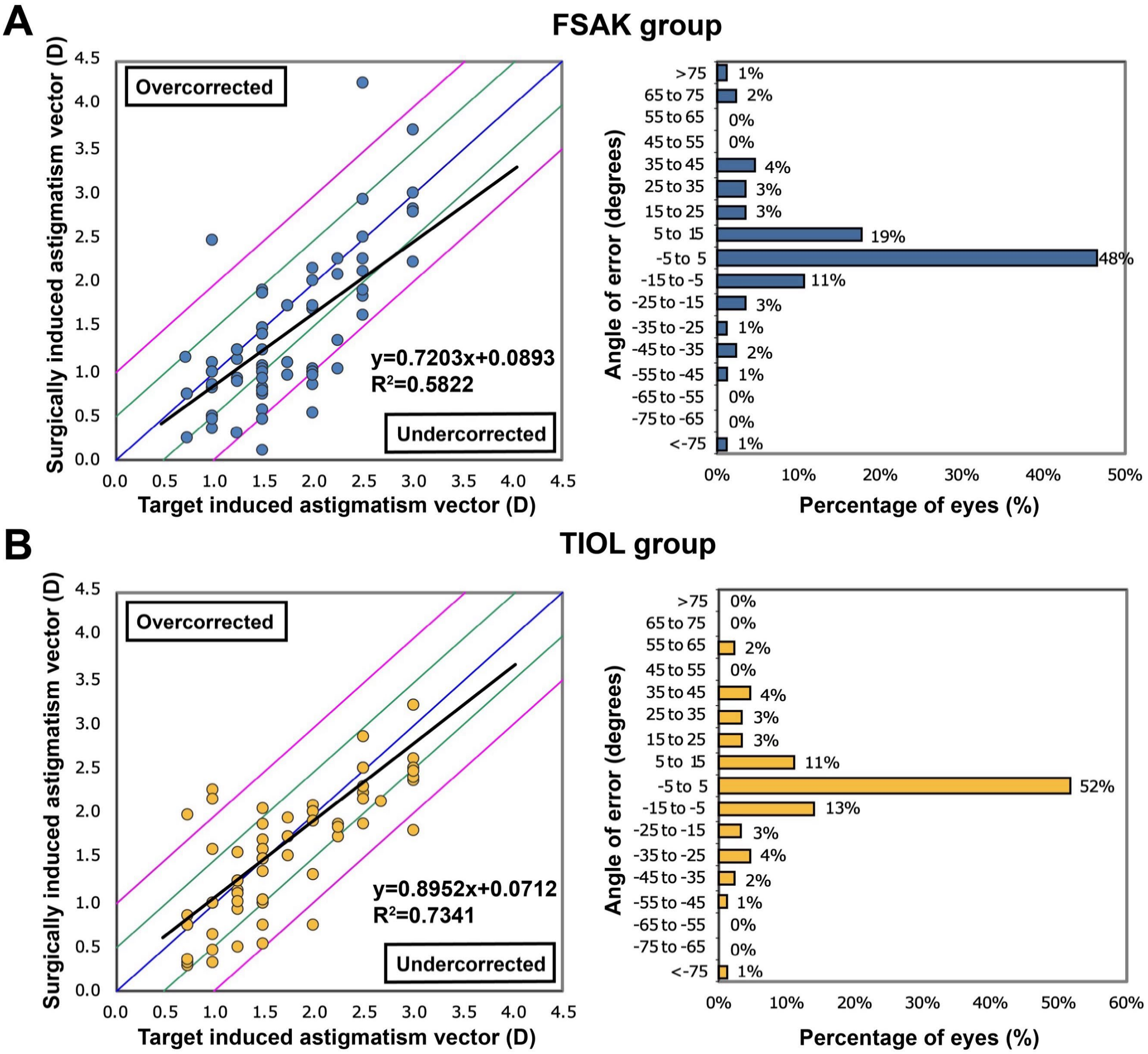

© 2025 Zhong Y et al. *JAMA Neurology*.

eFigure 3. A case of corneal perforation and anterior synechia in the FSAK group

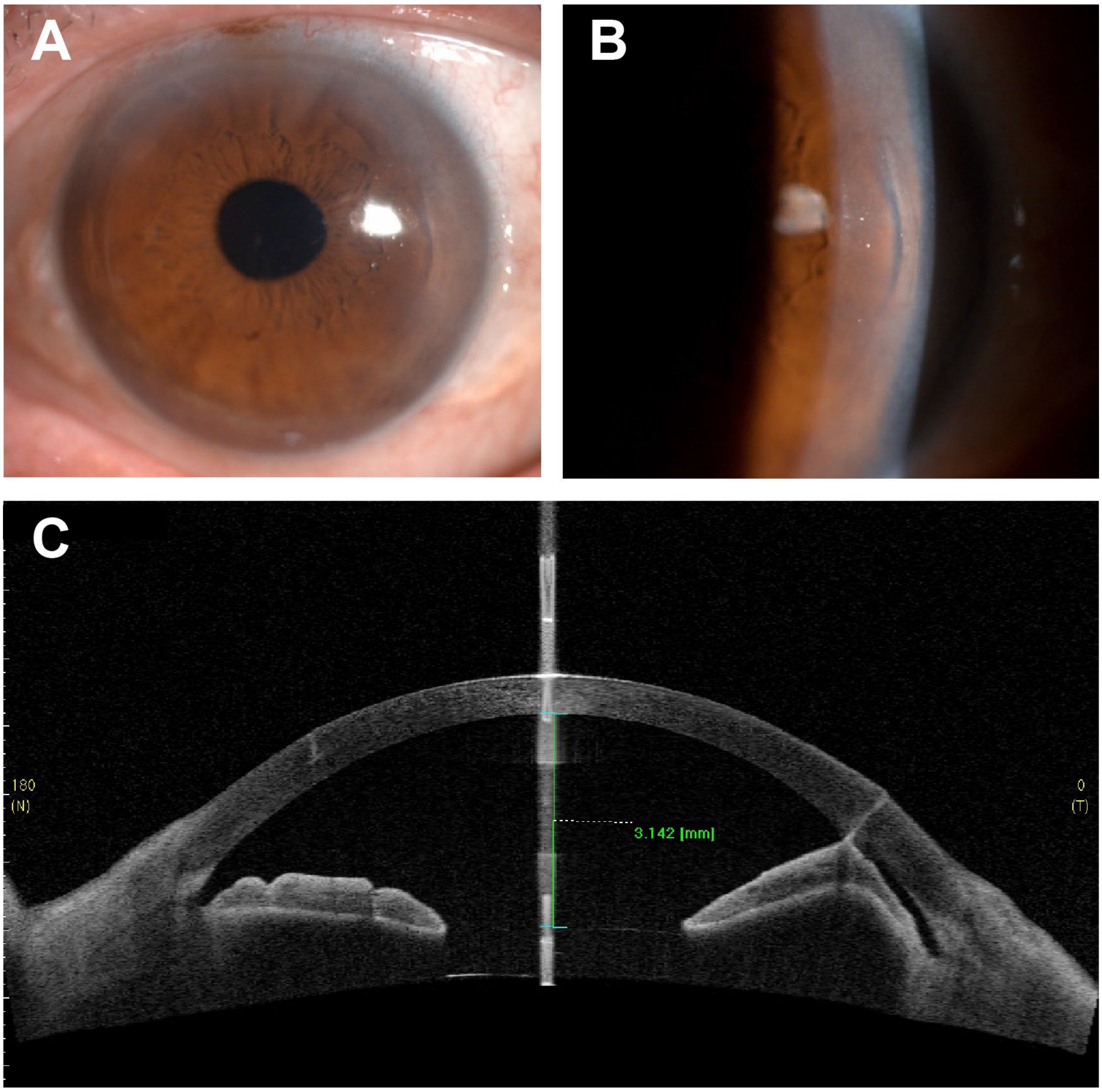

(A) Anterior segment photograph of the eye; (B) Magnified view illustrating the anterior synechia at the temporal arcuate incision; (C) Anterior segment optical coherence tomography (OCT) presenting the anterior synechia.

© 2025 Zhong Y et al. *JAMA Neurology*.
