# Supplementary material for: Femtosecond Laser Arcuate Keratotomy vs Toric Intraocular Lens Implantation in Cataract Surgery: A Randomized Clinical Trial
Source: JAMA Ophthalmol. 2025 Jan 23;143(3):199–206. doi: 10.1001/jamaophthalmol.2024.5887 (PMC11926645; doi:10.1001/jamaophthalmol.2024.5887)
Supplement: Supplement 4. — Data Sharing Statement. [file jamaophthalmol-e245887-s004.pdf]

Data Sharing Statement

Zhong. Femtosecond Laser Arcuate Keratotomy vs Toric Intraocular Lens Implantation in Cataract Surgery. *JAMA Ophthalmol*. Published January 23, 2025.
doi:10.1001/jamaophthalmol.2024.5887

Data
Additional Information: Chinese Clinical Trial Registry (ChiCTR.org.cn; identification number: ChiCTR2100051066)
Data available: No
